# Development of a High-Sensitivity Multiplex mRNA-Based KIT D816V Droplet Digital PCR Assay and Correlation with Tumor Load in Systemic Mastocytosis

**DOI:** 10.3390/ijms27146314

**Published:** 2026-07-16

**Authors:** Abdulrazzaq Alheraky, Arjan Simpelaar, Lucy B. Hesp, Albertus T. J. Wierenga, Moniek Heddema, Isidor Minović, Saskia K. Klein, Hanneke N. G. Oude Elberink, Ido P. Kema, André B. Mulder

**Affiliations:** 1 Department of Laboratory Medicine, University Medical Center Groningen, University of Groningen, P.O. Box 30001, 9700 RB Groningen, The Netherlands; a.a.alheraky@umcg.nl (A.A.); a.simpelaar@umcg.nl (A.S.); l.b.hesp@umcg.nl (L.B.H.); a.t.j.wierenga@umcg.nl (A.T.J.W.); m.heddema@umcg.nl (M.H.); i.minovic@umcg.nl (I.M.); i.p.kema@umcg.nl (I.P.K.); 2 Department of Hematology, University Medical Center Groningen, University of Groningen, P.O. Box 30001, 9700 RB Groningen, The Netherlands; s.k.klein@umcg.nl; 3 Department of Allergology, University Medical Center Groningen, University of Groningen, P.O. Box 30001, 9700 RB Groningen, The Netherlands; j.n.g.oude.elberink@umcg.nl; 4 Groningen Research Institute for Asthma and COPD (GRIAC), University Medical Center Groningen, University of Groningen, P.O. Box 30001, 9700 RB Groningen, The Netherlands

**Keywords:** systemic mastocytosis, mast cell, *KIT* D816V, droplet digital PCR, transcriptional activity, variant allele frequency, flowcytometry, tumor load

## Abstract

Detecting a *KIT* D816V mutation in patients with systemic mastocytosis (SM) needs a high-sensitivity assay. Additionally, high mutant *KIT* transcriptional activity and multilineage involvement are strong unfavorable prognostic parameters in SM. The current frequently used quantitative PCR-based *KIT* D816V assays have important analytical limitations, including considerable analytical variation in the low-concentration range, a need for a calibration curve, less multiplexing capabilities, and higher susceptibility to interference by PCR inhibitors. We developed, validated, and explored the diagnostic relevance of a novel multiplex *KIT* D816V cDNA droplet digital PCR (ddPCR), using *ABL1* and *KIT* as reference genes. Furthermore, we correlated the expressed allele burden (EAB) with variant allele frequency (VAF) and percentage of neoplastic mast cells (nMC%) in bone marrow samples of 79 SM patients. Our assay shows very low background signals and high assay precision, leading to a linear dynamic range spanning five orders of magnitude and very low limits of detection and quantification of 0.062% (EAB/*ABL1*) and 0.027% (EAB/*KIT*). The assay detects median 57 times more mutant cDNA copies than mutant gDNA copies in SM patients and positivity in 83% of SM-suspected patients with negative gDNA ddPCR results. Furthermore, correlations between EAB, VAF, and nMC% suggest the presence of *KIT* D816V in non-MCs, either with or without transcriptional activity. Using our novel high-sensitivity mRNA-based multiplex ddPCR *KIT* D816V assay returns high diagnostic yield in patients with suspicion for SM. We recommend incorporating our assay in routine SM diagnostics to determine low-burden *KIT* D816V.

## 1. Introduction

Mast cells (MCs) are hematopoietic cells of the innate immune system, primarily responsible for the IgE-mediated immune response, and they reside in several organs and tissues, including bone marrow (BM), the spleen, the liver, lymph nodes, and the gastrointestinal tract. A somatic point mutation in the *KIT* gene resulting in an aspartic acid-to-valine substitution at codon 816, i.e., *KIT* D816V, causes constitutive activity of the c-KIT receptor (CD117). In MCs, this leads to their increased survival and excessive proliferation, resulting in systemic mastocytosis (SM) [1]. The WHO classification of systemic MC disorders depends on the presence of so-called B (burden of disease) and C (cytoreduction required) findings and ranges from relatively mild variants, such as monoclonal MC activation syndrome (MMCAS), bone marrow mastocytosis (BMM, SM without skin lesions), indolent SM (ISM, SM with skin lesions) and smoldering SM (SSM), to advanced SM (AdvSM) including aggressive SM (ASM), mast cell leukemia (MCL), and SM with an associated hematological neoplasm (SM-AHN) [2].

The diagnosis of SM requires the assessment of pathologic MCs in BM and includes the detection of one of the most important diagnostic markers, i.e., the *KIT* D816V mutation [2]. However, very low numbers of *KIT* D816V-mutated BM MC often hamper the detection of this mutation [3,4], especially when performing the analysis of gDNA [5,6]. Given the strong cKIT (CD117) expression in MCs [7], several real-time qPCR assays have been developed for sensitive mRNA-based detection of *KIT* D816V [8,9,10,11]. However, qPCRs exhibit several important analytical disadvantages when compared to the droplet digital PCR (ddPCR) technique, like a larger analytical variation in the low-concentration range, the need for a calibration curve, less multiplexing capabilities with a higher chance of pipetting bias, and a higher susceptibility to interference by PCR inhibitors [12,13,14,15]. Moreover, the sensitivity of ddPCR assays can be readily improved by running additional wells and combining the results. The European Leukemia Net (ELN) has published recommendations for monitoring measurable residual disease (MRD) in patients with acute myeloid leukemia (AML) [16]. To achieve high-sensitivity MRD analysis, the ELN recommends molecular MRD assessment with a quantitative polymerase chain reaction (PCR) and the use of cDNA over gDNA for genes with high expression levels in AML cells, such as mutant Nucleophosmin 1 (*NPM1*). Furthermore, the ELN advises that transcript levels are normalized to the expression of the *ABL1* housekeeping gene. Because high analytical sensitivity is essential in SM diagnostics, we developed and validated an mRNA-based *KIT* D816V ddPCR assay with very high precision, sensitivity, and specificity. Furthermore, previously published qPCR assays used either the expression of the housekeeping *ABL1* gene or the expression of the wild-type *KIT* gene for normalization or as a reference gene [8,9,10,11]. To achieve the benefits of using both reference genes, we designed a multiplex ddPCR assay with both *ABL1* and *KIT as* reference genes.

Recently published data have revealed that high mutant *KIT* transcriptional activity and the presence of a *KIT* mutation in other non-MC myeloid and/or lymphoid cells, named multilineage involvement, seem to be strong unfavorable prognostic parameters in SM [17]. To enhance our understanding of *KIT* D816V transcriptional activity and patterns of clonal involvement, we correlated the transcriptional activity with the variant allele frequency (VAF) and actual percentage of neoplastic mast cells (nMC%) in bone marrow samples of SM patients.

## 2. Results

### 2.1. Assay Design

**Figure 1:**
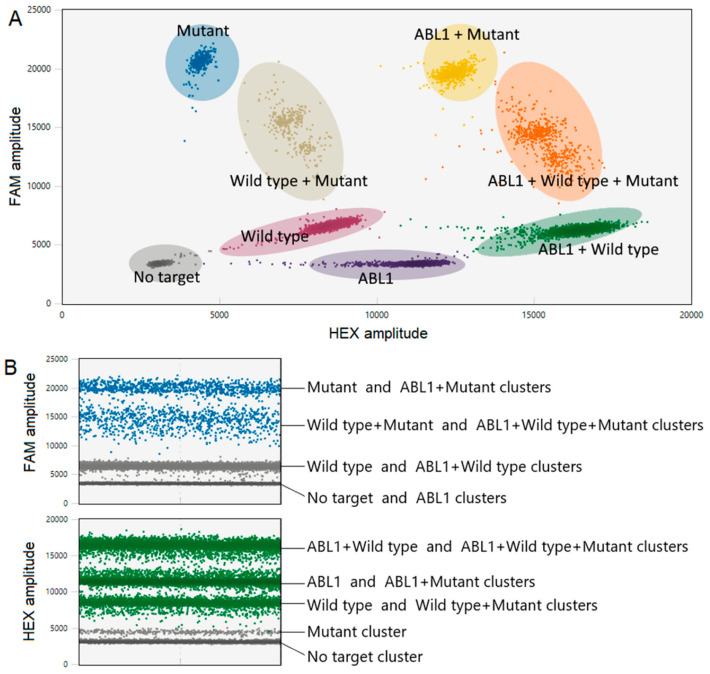
Representative fluorescence amplitude plots. A representative 2D fluorescence amplitude plot of a ddPCR analysis of a *KIT* D816V-positive sample (**A**). The black cluster on the plot represents the empty droplets, the blue cluster represents the droplets that are only positive for the *KIT* D816V mutant, the violet cluster represents the droplets that are only positive for the wild-type *KIT,* the purple cluster represents the droplets that are only positive for the *ABL1* reference target, the green cluster represents the droplets that are positive for both *ABL1* and wild-type *KIT*, the gray cluster represents the droplets that are positive for both the wild-type *KIT* and *KIT* D816V, the yellow cluster represents the droplets that are positive for both *ABL1* and *KIT D816V,* and the orange cluster represents the droplets that are positive for *ABL1, KIT D816V and KIT* wild-type. Representative 1D plots for all clusters (**B**). Results of *KIT* D816V mutant (blue FAM channel), *KIT* wild type (green HEX-LOW channel), and *ABL1* (green HEX-HIGH channel) are shown.

A multiplex *KIT* D816V ddPCR assay was developed to quantify *KIT* D816V expression levels normalized to both *ABL1* and *KIT* transcripts, enabling a highly accurate measurement of mutant *KIT* transcriptional activity and optimal comparison with mutant *KIT* VAF. A representative example of a 2D plot of a multiplex ddPCR reaction with clearly separated droplet clusters is shown in Figure 1A. The *KIT* wild-type and *ABL1* clusters are diagonally segregated and outlined with a pencil tool. High PCR efficiency was assessed by observing high consistency and segregation of clusters. The thresholds for the *KIT* D816V mutation detection (FAM signal), *KIT* wild-type detection (HEX-LOW signal), and *ABL1* detection (HEX-HIGH signal) are manually set to maximize the sensitivity and specificity. Slight fluorescence overlap between HEX and FAM channels improved the separation of the violet wild-type cluster and the green ABL1+ wild-type cluster from the purple ABL1 cluster, as also illustrated in 1D plots (Figure 1B).

### 2.2. Linearity and Dynamic Range

**Figure 2:**
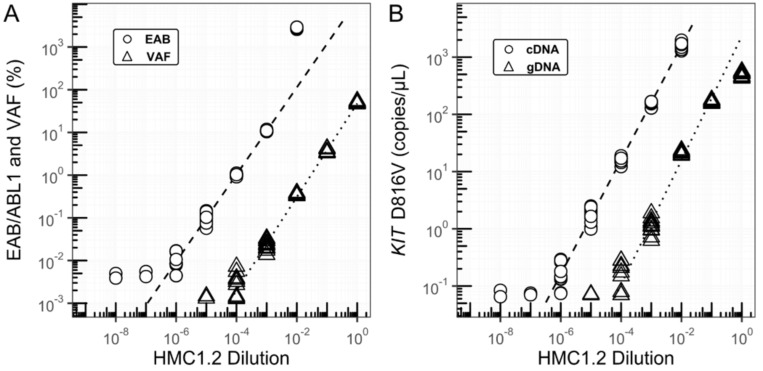
Linearity and dynamic range. Linearity and dynamic range of *KIT* D816V *ABL1*-normalized expressed allele burden (EAB/ABL1) and variant allele frequency (VAF) (**A**), and actual copy numbers (**B**) using HMC-1.2 serial dilutions. The undiluted and the 10^−1^ dilution samples were not included for the cDNA measurements because these dilutions oversaturated the ddPCR reaction. The dashed trendlines represent measurements of dilution series of HMC-1.2 cDNA, and the dotted trendlines represent measurements of dilution series of HMC-1.2 gDNA.

To determine the linearity and dynamic range, tenfold cDNA dilution series up to a 10^−8^ dilution from the human mast cell line 1.2 (HMC-1.2) [18], harboring a heterozygous *KIT* D816V mutation, were tested in duplicate at six different time points. The undiluted and 10^−1^ dilution samples were not included because these dilutions oversaturated the ddPCR reaction. The 10^−1^ and 10^−2^ dilution samples were diluted in DEPC H_2_O. All subsequent dilution steps were performed in cDNA from pooled *KIT* D816V-negative normal bone marrow (NBM) samples from healthy BM transplantation donors. The dilution series extended to positive *ABL1*-normalized *KIT* D816V values up to 10^−6^ dilution and was linear with an optimal *R*^2^ of 0.99 (Figure 2). The 10^−2^ dilution exhibited a slightly higher expressed allele burden (EAB) than the trend line, possibly due to a lower *ABL1* expression level in the HMC-1.2 cell line.

In addition, tenfold serial dilutions of HMC-1.2 gDNA in normal bone marrow (NBM) gDNA, ranging from undiluted (10^0^) to a 10^−6^ dilution, were tested in duplicate at six different time points (Figure 2). The dynamic range of the VAF analysis spanned five orders of magnitude, ranging from undiluted to a 10^−4^ dilution, with an optimal *R*^2^ linearity of 0.99.

### 2.3. Analytical Sensitivity and Specificity

The limit of blank (LoB) was estimated by measuring six cDNA NBM samples at ten different time points and was calculated at a 95% confidence level as the mean value + 1.645 × standard deviation (SD). The LoB was 0.007% EAB when using *ABL1* as a reference gene and 0.013% EAB when using *KIT* as a reference gene (Table 1).

For calculating the limit of detection (LoD), aliquots from the six split NBM samples were spiked with the smallest possible amount of HMC-1.2 that returned a positive *KIT* D816V signal higher than the LoB in more than 95% of measurements, which was determined as the 2 × 10^−6^ HMC-1.2 dilution. These low-concentration samples were measured in parallel with the *KIT* D816V-negative samples. The LoD was calculated with a 95% confidence level as the LoB + 1.645 × SD of the low concentration sample and turned out to be 0.062% EAB when using *ABL1* as the reference gene and 0.027% EAB when using *KIT* as the reference gene. The limit of quantification (LoQ) was defined as the concentration where the inter-assay coefficient of variation (CV) equals 35%.

The inter-assay CV of the low-concentration sample used for establishing the LoD, i.e., the 2 × 10^−6^ HMC-1.2 dilution, was 24.1% when using *ABL1* as the reference gene and 34.1% when using *KIT* as the reference gene (Table 2). Because the LoDs exhibited CVs lower than 35%, the LoQs were defined as equal to the LoDs. The LoB, LoD, LoQ, and intra- and inter-assay CV values of the ddPCR gDNA assay were also determined by similar procedures. The inter-assay CV of the low-concentration sample used for establishing the LoD, i.e., the 1 × 10^−3^ HMC-1.2 dilution, yielded VAF results that were comparable to the results found previously [19], and comparable to our cDNA assay, with an LoB, LoD, and LoQ of 0.002%, 0.024%, and 0.041%, respectively (Table 1).

### 2.4. Normalization to ABL1 Versus KIT

**Figure 3:**
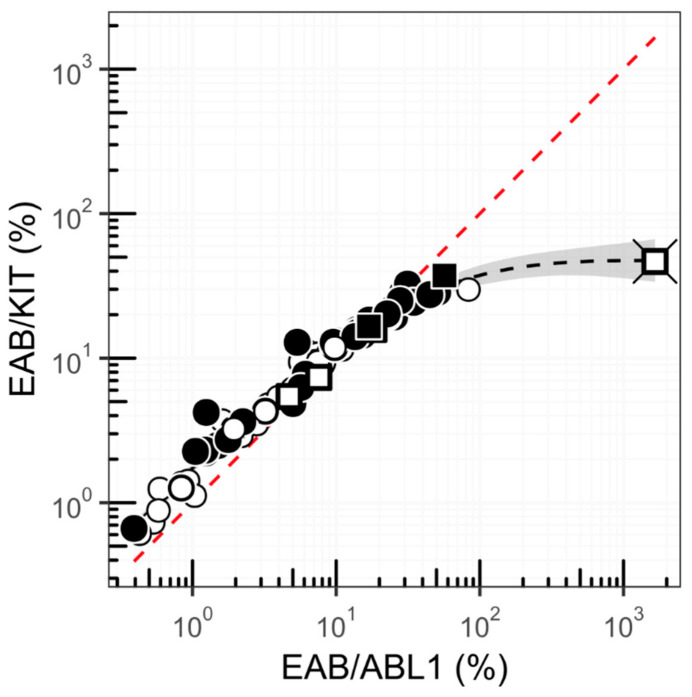
Normalization with ABL1 versus KIT reference gene. Correlation (black dashed line) between ABL1-normalized KIT D816V expression (KIT D816V/ABL1) versus total KIT-normalized KIT D816V expression (KIT D816V/KIT total) in 73 patients with an ISM (circles) and six patients with an AdvSM (squares). The red dashed line follows y = x. Abbreviation: EAB (expressed allele burden).

The choice of reference gene impacts the accuracy and precision of the *KIT* D816V quantification. Therefore, we compared the use of *ABL1* versus total *KIT* as reference genes across SM patient samples (Figure 3). Both methods revealed a nonlinear correlation, in which a low tumor load (<0.1%) resulted in a trend toward slightly higher total *KIT*-normalized values, whereas a higher load showed higher *ABL1*-normalized results.

### 2.5. Sensitivity of cDNA Versus gDNA Analyses

**Figure 4:**
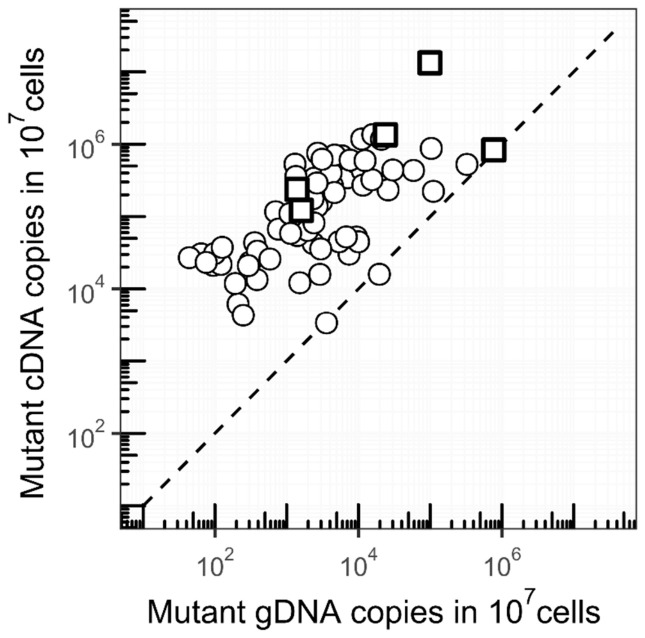
Sensitivity of cDNA versus gDNA analyses for KIT D816V detection in bone marrow (BM). The abundance of KIT D816V mutant cDNA and gDNA copies in split BM samples (10^7^ leukocytes) from 73 patients with an ISM (circles) and six patients with an AdvSM (squares). The dashed line follows y = x.

The abundance of *KIT* D816V mutant cDNA and gDNA copies in split BM samples (10^7^ cells) from a group of 73 patients with ISM and six patients with advanced SM (AdvSM) was compared (Figure 4). The median number of mutant cDNA copies was 57 (range 1–614) times higher than mutant gDNA copies. Higher mutant abundance when using RNA instead of DNA samples increases detection probability and sensitivity, especially in cases with a low mutant burden where detection is affected by stochastic effects.

To illustrate the impact of using our high-sensitivity *KIT* D816V cDNA ddPCR assay, samples from a group of 18 patients with a high suspicion for SM, but insufficient diagnostic criteria to establish the diagnosis of SM, and negative *KIT* D816V gDNA ddPCR results were evaluated. Fifteen out of 18 patients with negative *KIT* D816V gDNA ddPCR results demonstrated positive *KIT* D816V expression levels, with *KIT* EAB/*ABL1* ranging from 0.021% to 0.865%, and *KIT* EAB/KIT ranging from 0.028% to 0.632%. Seven out of 15 patients also showed MCs with aberrant CD25 expression, supporting the presence of abnormal MCs. Constantly negative no-template control (NTC) values and no-amplification control (NAC) values below the LoB made the possibility of false-positive background signals unlikely. So, the cDNA ddPCR analysis detected *KIT* D816V expression in 83% (15/18) of SM-suspected patients with negative gDNA ddPCR results.

### 2.6. Correlations Between KIT D816V VAF, KIT D816V EAB and nMC%

**Figure 5:**
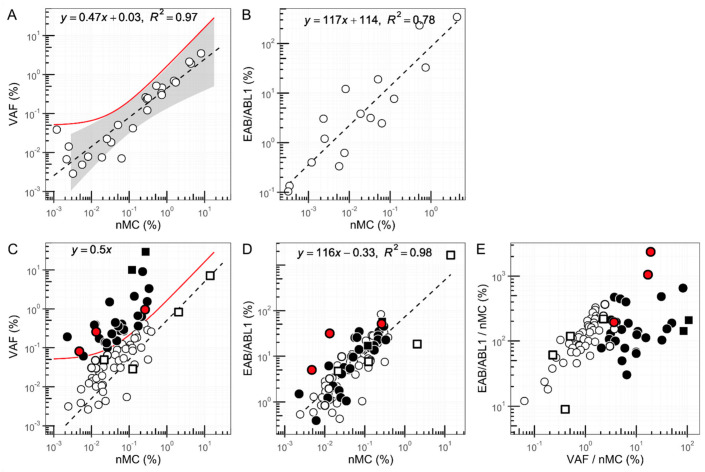
Correlations between KIT D816V VAF, KIT D816V EAB, and nMC%. Correlation between the *KIT* D816V VAF and nMC% in HMC-1.2 gDNA tenfold dilution series in gDNA from pooled NBM samples (**A**). The undiluted sample was not included because this sample oversaturated the ddPCR reaction. The dashed black line follows the trendline y = 0.47x + 0.03. The correlation is shown with a confidence interval covering the measurement range of 99.999%. The solid red line corresponds to the upper boundary of this range and is used as a cut-off value for detecting non-MC-restricted involvement of *KIT* D816V. Correlation between the *KIT* D816V EAB/*ABL1* and nMC% in HMC-1.2 cDNA tenfold dilution series in cDNA from pooled NBM samples. (**B**). The undiluted and 10% dilution samples were not included in the cDNA assay because these samples oversaturated the ddPCR reaction. The dashed black line follows the trendline y = 117x + 114. Correlation between the VAF and the nMC% in BM samples of 73 patients with an ISM (circles) and six patients with an AdvSM (squares) (**C**). The cut-off value for detecting non-MC-restricted involvement of *KIT* D816V is indicated as a solid red line. Patients who showed higher VAFs than expected relative to the nMC% are indicated with filled circles (ISM patients) or filled squares (AdvSM patients). The dashed black line follows y = 0.5x. Correlations between the *KIT* D816V EAB/*ABL1* and the nMC% in BM samples of 73 patients with an ISM (circles) and six patients with an AdvSM (squares) (**D**). The dashed black line follows the trendline y = 116x − 0.33. Correlation between the *KIT* D816V EAB/*ABL1* (*KIT* D816V EAB/nMC%) and the *KIT* D816V VAF (*KIT* D816V VAF/nMC%) after normalizing both for the nMC% (**E**). Results from three ISM patients with a B-finding are indicated in red. VAF: variant allele fraction, EAB: expressed allele. burden, nMC%: percentage of neoplastic mast cells in bone marrow.

We also correlated the *KIT* D816V EAB and VAF with the actual percentage of neoplastic BM MCs (nMC%). Firstly, we determined the analytical variance in the correlation results of the flow cytometric and PCR analyses by testing tenfold HMC-1.2 gDNA and cDNA dilution series up to 10^−3^ dilutions in duplicate (Figure 5A,B). The *KIT* D816V VAF results showed good agreement with the expected distribution around 0.5 times the nMC%, as aberrant MCs exhibit one heterozygous *KIT* D816V allele and one *KIT* wild-type allele, with an *R*^2^ of 0.97 (Figure 5A). The upper limit values of this correlation were defined as the cut-off value for detecting non-MC-restricted *KIT* D816V. Of note, at lower nMC%, the upper bound was set at a relatively higher VAF value due to higher analytical variance. In addition, the *KIT* D816V EAB showed more than 100-fold higher values than the nMC%, with a good correlation with an *R*^2^ of 0.78 (Figure 5B).

Next, we tested bone marrow samples of 79 SM patients and also found a positive correlation between the VAF and nMC% (Spearman ρ = 0.64, *p* < 0.01) (Figure 5C). However, 28 out of 73 patients with ISM and two out of six patients with AdvSM showed VAFs higher than 0.5 times the nMC%, suggesting that other non-MC BM cells also harbored the *KIT* D816V mutation, i.e., possible presence of multilineage involvement. Further, the *KIT* D816V EAB showed a distribution around about 100 times the nMC% and a strong correlation with an *R*^2^ of 0.98 (Zou’s 95% confidence interval: 0.06–0.29; *p* < 0.01) (Figure 5D). Notably, even most patients with a presumed non-MC-restricted involvement had correlations between the *KIT* D816V EAB and nMC% that did not deviate from the main trendline.

Finally, we correlated the EAB and the VAF after correction for the nMC% (Figure 5E). As expected, patients with VAFs around 0.5 times the nMC% showed EAB/nMC% ratios of about 100, comparable to the transcriptional activity of the HMC-1.2 cells. Additionally, most patients with VAFs higher than 0.5 times the nMC% demonstrated EAB/nMC% ratios also around 100. However, a few patients with VAFs higher than 0.5 times showed much higher EAB/nMC% ratios, and the two patients with the highest EAB/nMC% ratios (2.358% and 1.052%) exhibited an ISM with a B-finding, while only one out of the remaining 71 ISM patients had a B-finding (Figure 5E).

## 3. Discussion

Here we present, for the first time, a multiplex mRNA-based *KIT* D816V ddPCR assay that covers both *ABL1* and *KIT* wild-type reference genes, enabling highly accurate measurement of mutant *KIT* transcriptional activity leading to a very high diagnostic yield. Our assay shows extremely low background signals with LoBs of 0.007% (EAB/*ABL1*) and 0.013% (EAB/*KIT*) and a high assay precision, leading to very low LoD/LoQ values of 0.062% (EAB/*ABL1*) and 0.027% (EAB/*KIT*). The linear dynamic range spans five orders of magnitude with a slope of 0.999 and an *R*^2^ of 0.99, indicating excellent PCR efficiency. We employed a ddPCR method instead of the conventionally used real-time qPCR because of the analytical advantages of direct and accurate quantification without the need for a calibration curve, the multiplexing capacity with reduced pipetting bias, and a lower impact of PCR inhibitors [12,13,14,15]. Moreover, using the same analytical ddPCR technique, i.e., our mRNA-based ddPCR assay and a widely used and extensively validated DNA-based ddPCR assay [19], enabled an optimal comparison between *KIT* D816V transcriptional activity and VAF. However, although ddPCR provides several analytical advantages over qPCR, its broader implementation in routine clinical diagnostics may be limited by higher reagent and operational costs, requirement for specialized instrumentation and technical expertise, and its currently lower availability in routine diagnostic laboratories compared with qPCR-based platforms.

We designed a multiplex ddPCR assay with both *ABL1* and *KIT* as reference genes to achieve the benefits of using both reference genes. For exact quantification, a reference gene expressed by all cells, such as the *ABL1* housekeeping gene, is required. Furthermore, including the *KIT* wild-type probe in the assay reduces possible non-specific binding of the *KIT* D816V probe to the wild-type allele. Moreover, our assay offers the opportunity to compare results with data from other studies that either used *ABL1* or *KIT* as a reference gene [8,9,10,11]. Finally, the use of a *KIT* reference facilitates the optimal comparison between mutant *KIT* expression and VAF signals [20]. To achieve high sensitivity, maximum input of *KIT* D816V transcripts without oversaturation of the ddPCR reaction is needed [21]. Clonal MCs express high amounts of heterozygous *KIT* D816V and *KIT* wild-type transcripts and relatively low numbers of *ABL1* transcripts. When using only one reference gene in the assay, *ABL1* offers the advantage of having less impact on the risk of oversaturation and therefore enables higher cDNA input in the assay, with the possibility to detect very low numbers of mutant *KIT* transcripts. In addition, using *KIT* as the only reference gene also enables high sample input, because only signals from *KIT*-expressing CD117-positive cells, including MCs, myeloid blasts, and promyelocytes, are measured.

However, despite all these advantages, there are some drawbacks. Firstly, the use of a second reference gene impairs the maximum sample input, although this effect seems to be limited. The only extra positive droplets in the assay are the droplets that are only positive for the second reference gene, and a high sample input gives relatively fewer droplets with copies of only one gene. Additionally, *ABL1*-normalized *KIT* expression results might be influenced by variable levels of *ABL1* expression [21], potentially causing a risk of falsely increased or decreased *KIT* D816V expression values.

Furthermore, we compared the results of our mRNA-based ddPCR assay with the results of the commercially available and widely used gDNA-based ddPCR [18]. Although both assays demonstrated similar high analytical performances, the cDNA measurements proved superior to the gDNA results, as observed by finding a median 57-fold higher abundance of mutant *KIT* cDNA transcripts compared to mutant *KIT* gDNA alleles in BM samples of SM patients. The diagnostic impact of the much higher sensitivity is evident, as our data showed that the cDNA assay was able to detect *KIT* D816V transcripts in 83% of the patients with a clinical suspicion for SM but negative *KIT* gDNA ddPCR results. Taken together, these findings demonstrate that our novel cDNA ddPCR assay detects *KIT* D816V mutants with significantly higher sensitivity in BM samples than the gDNA ddPCR assay, while retaining high specificity.

To evaluate the relevance of measuring *KIT* transcriptional activity to better understand patterns of clonal mutant *KIT* involvement, we correlated the transcriptional activity with the VAF and the nMC% and found three different types of patients. First, 49 out of 79 (62%) patients, including four patients with an AdvSM, showed VAFs around 0.5 times the nMC% and EAB/nMC% ratios comparable to the transcriptional activity of HMC-1.2 cells, indicating MC-restricted *KIT* D816V expression. Second, most remaining patients, including two with an AdvSM, demonstrated VAFs higher than 0.5 times the nMC%, but the EAB/nMC% ratios were still comparable with the transcriptional activity of HMC-1.2 cells, suggesting that in these patients, non-MCs also carry the *KIT* D816V mutation in their DNA (i.e., possible presence of multilineage involvement), but have no transcriptional activity. These findings are in line with data from Escribano et al. [22] and Teodosio et al. [23], demonstrating multilineage involvement in 9.5% and 30% SM patients, respectively, and with results from several other studies showing multilineage involvement in CD117-negative cells, such as granulocytes, monocytes, and lymphocytes [23,24,25]. However, the interpretation of multilineage *KIT* D816V involvement should be done carefully because it remains indirect, i.e., no sorted-cell or single-cell confirmation was performed. Moreover, alternative explanations, such as the *KIT* D816V copy number gain or loss of heterozygosity or the *KIT* wild-type copy number loss, were not excluded. Finally, some patients with suggested multilineage involvement showed clearly higher EAB/nMC% ratios, which might suggest that the *KIT* D816V mutation is not only present in the DNA but also expressed in non-MCs, such as CD117-positive early pluripotent progenitor cells, myeloblasts, and promyelocytes [26]. Remarkably, the two patients with the highest EAB/nMC% ratios both exhibited an ISM with a B-finding, i.e., one had hepatomegaly, and the other one had splenomegaly, while only one out of the remaining 71 ISM patients had a B-finding. These findings seem to be in line with data, showing that ISM patients who evolve to aggressive disease subtypes harbor the *KIT* D816V mutation in early CD117-positive hematopoietic precursors [22,23,24,25]. However, other possible explanations, like increased *KIT* D816V transcription in MCs or variable RNA integrity, can give the same results and were not excluded. Therefore, there is far too little data available to claim this association. This observation must be interpreted as preliminary and hypothesis-generating. So, our findings suggest that increased VAF/nMC% and EAB/nMC% ratios might possibly be used as surrogate markers for multilineage involvement of *KIT* mutations in SM patients. This is of special relevance because complex and not widely available cell sorting techniques are required to determine mutant *KIT* multilineage involvement.

Our study has some limitations. Samples were primarily collected for clinical testing, and the inclusion of patients and data was performed retrospectively. This resulted in limited sample volumes being available for analyses, restricting some aspects of the analytical and clinical validations, including meaningful statistical testing in some small subgroups and evaluation of our assay in a large number of patients. In addition, no cell sorting analyses to confirm the presence of multilineage involvement or testing possible other explanations were performed due to the retrospective nature of our study. Consequently, further clinical multicenter validation studies in larger patient cohorts, including cell sorting experiments, are needed to confirm our findings concerning the relevance of correlating *KIT* D816V transcriptional activity with VAF and tumor load in the routine clinical practice of SM patients.

## 4. Materials and Methods

### 4.1. KIT D816V cDNA Droplet Digital PCR Assay Design

A multiplex ddPCR assay was developed to quantify *KIT* D816V (NM_000222.2, GRCh37 chromosome 4: 55,524,085-55,606,881) expression, together with *KIT* wild-type and *ABL1* (NM_007313.2, GRCh37 chromosome 9: 133,589,333-133,763,062) reference transcripts, as was done before for different Nucleophosmin 1 (*NPM1*) mutations [27]. For the measurement of *KIT* expression, primers and a FAM (Fluorescein)-labeled *KIT* D816V probe and a HEX (Hexachloro-Fluorescein)-labeled *KIT* wild-type probe were designed (Supplementary Materials, Selection of primers and probes and Table S1). For measurement of reference *ABL1* transcripts, a commercially available mix of primers and a HEX-labeled probe (Bio-Rad Laboratories, Hercules, CA, USA) was included in the assay. The optimal annealing temperature was determined at 57 °C by testing a temperature gradient (Supplementary Materials, Selection of optimal annealing temperature and Figure S1).

### 4.2. Droplet Digital PCR Protocols

*KIT* D816V cDNA levels were analyzed using an optimized ddPCR protocol. Briefly, the PCR mix contained ddPCR Multiplex Supermix, *KIT* primers, a *KIT* D816V probe and a *KIT* wild-type probe, and *ABL1* primers and probe. Data were analyzed on a QX200 droplet reader (Bio-Rad Laboratories, Hercules, CA, USA) with QX manager or Quantasoft Analysis Pro software (version 1.0.596) to distinguish *KIT* D816V (FAM), *KIT* wild-type (HEX-LOW), and *ABL1* (HEX-HIGH) signals. A detailed protocol is described in the Supplementary Materials (Supplementary Materials, *KIT* D816V Droplet Digital PCR Protocol and Table S2). Results were expressed as a fraction (%) normalized to *ABL1* reference gene expression (*KIT* D816V copies divided by *ABL1* copies × 100%), or as a fraction (%) normalized to the total *KIT* expression (*KIT* D816V copies divided by *KIT* D816V copies + *KIT* wild-type copies × 100%).

For *KIT* D816V gDNA analysis, a commercially available and extensively validated ddPCR assay [19] was purchased from Bio-Rad Laboratories (Hercules, CA, USA) and used according to the manufacturer’s recommendations. Results were expressed as variant allele frequency (VAF), i.e., the percentage mutated *KIT* D816V copies divided by the total number of *KIT* copies × 100%.

### 4.3. Study Samples

For analytical validation experiments, RNA and gDNA from the human mast cell line 1.2 (HMC-1.2) [18], harboring a heterozygous *KIT* D816V mutation, and normal bone marrow (NBM) samples from healthy BM transplantation donors, were used. For testing clinical utility, BM RNA and gDNA samples from patients referred to the University Medical Center of Groningen between 2014 and 2024 were selected, based on symptoms of MC activation or established SM diagnosis. SM diagnosis and classification were established according to the World Health Organization (WHO) criteria for SM, as previously described [28,29]. Samples for this study were retrospectively included if flow cytometric results and split RNA and gDNA samples were available to perform the analyses needed for this study. RNA and gDNA BM samples from 18 individuals with a high suspicion of SM but negative gDNA *KIT* D816V ddPCR results were selected to assess the impact of using the highly sensitive assay on diagnostic accuracy. Also, RNA and gDNA BM samples from 73 patients with indolent SM (ISM) and from 6 patients with advanced SM (AdvSM) were processed for PCR analyses to determine the EAB and VAF levels. Further details about sample collection, RNA and gDNA isolation, and cDNA synthesis are described in the Supplementary Materials section (Supplementary Materials, Sample collection, RNA and gDNA isolation, and cDNA synthesis and Tables S3 and S4).

### 4.4. Flow Cytometric Analysis

Flow cytometric analysis was performed in all patient samples as part of the routine diagnostic procedure. The actual percentage of neoplastic MCs in BM (nMC%) was defined as the population of CD25-positive MCs using all CD45-positve BM cells as the denominator. Further details on the flow cytometric analysis have been described previously [30].

### 4.5. Analytical Assay Validation

The *KIT* D816V mRNA ddPCR assay was validated in accordance with the EP05 and EP17 guidelines of the Clinical and Laboratory Standards Institute (CLSI) and the Digital MIQE guidelines [31,32,33]. The analytical sensitivity and specificity were determined using the limit of blank (LoB), limit of detection (LoD), and limit of quantification (LoQ). Intra- and inter-assay variability was quantified with the coefficients of variation (CV). Finally, the performance of our new assay was compared with that of a widely used and commercially available *KIT* D816V gDNA ddPCR assay [19] and a qPCR previously used for routine clinical diagnostics (Supplementary Materials, Method comparison between ddPCR and qPCR and Figure S2).

### 4.6. Statistical Analysis

The significance of correlations was determined using Spearman’s rank correlation coefficient (ρ) due to non-normal and/or skewed distributions. Differences between correlations were evaluated using Zou’s 95% confidence interval method [34]. All distributions were tested for normality using Q-Q plots. All statistical analyses and graphical representations were performed using RStudio version 1.4.1106 (PBC).

## 5. Conclusions

We present the development, validation, and exploratory clinical evaluation of a high-sensitivity multiplex mRNA-based KIT D816V ddPCR assay. Implementation of this highly sensitive assay in the daily clinical practice of SM will increase the diagnostic yield in bone marrow cells, especially when no *KIT* mutation is found with DNA assays. Furthermore, correlating *KIT* D816V transcriptional activity with VAF and mast cell tumor load might show promise as a surrogate approach for identifying multilineage *KIT* mutation involvement, pending confirmation by cell-sorting or single-cell investigations. Additionally, further prospective multicenter clinical validation studies in larger patient cohorts are needed to prove its possible value in risk assessment.

## Figures and Tables

**Table 1:** Analytical sensitivity and specificity parameters of the cDNA and gDNA assay and the HMC-1.2 results.

	gDNA	cDNA	
	VAF (%)	EAB/ABL1 (%)	EAB/KIT (%)
Limit of blank (LoB)	0.002	0.007	0.013
Limit of detection (LoD)	0.024	0.062	0.027
Limit of quantification (LoQ)	0.041	0.062 ^1^	0.027 ^2^
HMC-1.2 undiluted	49.70	1917.34	49.99

The VAF was calculated as the percentage mutated *KIT* D816V copies divided by the total number of *KIT* copies × 100%, using gDNA from NBM samples (LoB) or 1 × 10^−3^ HMC-1.2 dilution samples (LoD/LoQ). The EAB/ABL1 was calculated as a fraction (%) normalized to *ABL1* reference gene expression (*KIT* D816V copies divided by *ABL1* copies × 100%). The EAB/KIT was calculated as a fraction (%) normalized to the total *KIT* expression (*KIT* D816V copies divided by *KIT* D816V copies + *KIT* wild-type copies × 100%). For EAB calculations, cDNA from NBM samples (LoB) or 2 × 10^−6^ HMC-1.2 dilution samples (LoD/LoQ) were used. ^1,2^ LoDs exhibited inter-assay CVs lower than 35%; therefore, the LoQs were defined as equal to the LoDs. Abbreviations: VAF (variant allele frequency), EAB/ABL1 (ABL1-normalized expressed allele burden), EAB/KIT (KIT-normalized expressed allele burden), NBM (normal bone marrow), HMC-1.2 (human mast cell line 1.2).

**Table 2:** Intra-assay and inter-assay variation (CV%) of the cDNA and gDNA assay.

	gDNA VAF (%)		cDNA EAB/ABL1 (%)		cDNA EAB/KIT (%)	
	Intra-Assay (CV%)	Inter-Assay (CV%)	Intra-Assay (CV%)	Inter-Assay (CV%)	Intra-Assay (CV%)	Inter-Assay (CV%)
HMC-1.2 × 10^0^	3.26	4.78	-	-	-	-
HMC-1.2 × 10^−1^	3.59	5.61	-	-	-	-
HMC-1.2 × 10^−2^	4.10	6.00	1.28	3.50	0.46	0.81
HMC-1.2 × 10^−3^	12.2	24.3	1.43	3.53	1.56	3.30
HMC-1.2 × 10^−4^	74.9	87.2	3.92	6.44	4.02	6.35
HMC-1.2 × 10^−5^	100	224.1	14.6	26.3	14.27	26.2
HMC-1.2 × 2 × 10^−6^	-	-	15.7	24.1	17.1	34.1
HMC-1.2 × 10^−6^	-	-	42.7	53.0	29.5	53.4
HMC-1.2 × 10^−7^	-	-	100	226.0	100	225.9
HMC-1.2 × 10^−8^	-	-	100	225.6	100	225.9

The VAF was calculated as the percentage mutated *KIT* D816V copies divided by the total number of *KIT* copies × 100%, using gDNA from NBM samples (LoB) or 1 × 10^−3^ HMC-1.2 dilution samples (LoD/LoQ). The EAB/ABL1 was calculated as a fraction (%) normalized to *ABL1* reference gene expression (*KIT* D816V copies divided by *ABL1* copies × 100%). The EAB/KIT was calculated as a fraction (%) normalized to the total *KIT* expression (*KIT* D816V copies divided by *KIT* D816V copies + *KIT* wild-type copies × 100%). For EAB calculations, cDNA from NBM samples (LoB) or 2 × 10^−6^ HMC-1.2 dilution samples (LoD/LoQ) were used. Abbreviations: CV% (coefficient of variation in %), VAF (variant allele frequency), EAB/ABL1 (ABL1-normalized expressed allele burden), EAB/KIT (KIT-normalized expressed allele burden), NBM (normal bone marrow), HMC-1.2 (human mast cell line 1.2).

## Data Availability

The data that support the findings of this study are available from the corresponding author upon reasonable request. Not all supporting data are publicly available due to privacy restrictions.

## Supplementary Materials

The following supporting information can be downloaded at https://www.mdpi.com/article/10.3390/ijms27146314/s1.
