# Supplementary material for: Development of a High-Sensitivity Multiplex mRNA-Based KIT D816V Droplet Digital PCR Assay and Correlation with Tumor Load in Systemic Mastocytosis
Source: Int J Mol Sci. 2026 Jul 16;27(14):6314. doi: 10.3390/ijms27146314 (PMC13410330; doi:10.3390/ijms27146314)
Supplement: Supplementary file 1 [file ijms-27-06314-s001.zip › ijms-4336285-supplementary.pdf]

Supplementary Materials

1. Assay design

1.1 Selection of primers and probes……………………………….……………………1

1.2 Selection of optimal annealing temperature………………….……………………….2

2. KIT D816V droplet digital PCR protocol……………………...…………………………3

3. Sample collection, RNA and gDNA isolation and cDNA synthesis……………………...….5

4. Method comparison between ddPCR and qPCR…..………………………….……………8

References……………………………………………………………………………....9

1. Assay design

1.1 Selection of primers and probes

The multiplex mRNA-based *KIT* D816V Droplet Digital PCR assay was designed so that a FAM (6-Carboxy-Fluorescein)-labeled probe recognizes the *KIT* D816V mutation and a HEX (Hexachloro-Fluorescein)-labeled probe recognizes the *KIT* wild-type (NM_000222.2, GRCh37 chromosome 4: 55,524,085-55,606,881). Primers and probe were designed using Primer-BLAST (National Center for Biotechnology Information, Bethesda, MD, USA), which automatically checks for specificity across the genome and flags potential off-target binding. To further decrease the possibility of mispriming, primers were designed to span an exon-exon junction. Primers and probes were obtained from Eurofins Genomics (Ebersberg, Germany). To increase the specificities, minor groove binding (MGB) probes containing locked nucleic acid (LNA) modifications were selected (Table S1). To avoid mismatches under the primers and probes, we verified the absence of SNPs or other common variants under the primer and probe binding regions with the UCSC genome browser (http://www.genome.ucsc.edu) using the dbSNP release 155 database. For measurement of *ABL1* (NM_007313.2, GRCh37 chromosome 9: 133,589,333-133,763,062) reference cDNA transcripts, a commercially available mix of primers and HEX-labeled probe obtained from Bio-Rad Laboratories, Hercules, CA, USA, catalogue number: dHsaCPE5046927 was included in the assay.

**Table S1.** Nucleotide sequences of *KIT* primers and probes.	
**Primer/probe**	**Sequence (5'-3')**
***KIT* wild-type probe**	HEX-AGCCAGA+G+A+CATCAAGAAT-MGB
***KIT* D816V probe**	FAM-AGCCAGA+G+**T**+CATCAAGAAT-MGB
***KIT* forward primer**	CCTCCAAGAATTGTATTCAC
***KIT* reverse primer**	AGCGTTTCCTTTAACCAC

Table S1: *Nucleotide sequences of KIT primers and probes*. The mismatching nucleotide to the wild type *KIT* sequence (resulting in an adenine by thymine substitution at position 2447) in the *KIT* D816V probe is shown in bold. The forward primer binding starts at c.2409 and ends at c.2428, the reverse primer binding starts at c.2545 and ends at c.2528 (amplicon length 137 bp). To increase specificity, locked nucleic acid (LNA)–modified minor groove binding (MGB) probes were selected.

1.2 Selection of optimal annealing temperature

An annealing temperature gradient, ranging from 55°C to 65°C was used to find the optimal annealing temperature. An annealing temperature of 57 °C was found to be the optimal annealing temperature, resulting in a high amplitude of the positive signal when using cDNA derived from a HMC-1.2 10^{-3} dilution (A) and from a SM patient (B), and almost no nonspecific background amplification was seen when using normal bone marrow (NBM) derived cDNA (Figure S1).

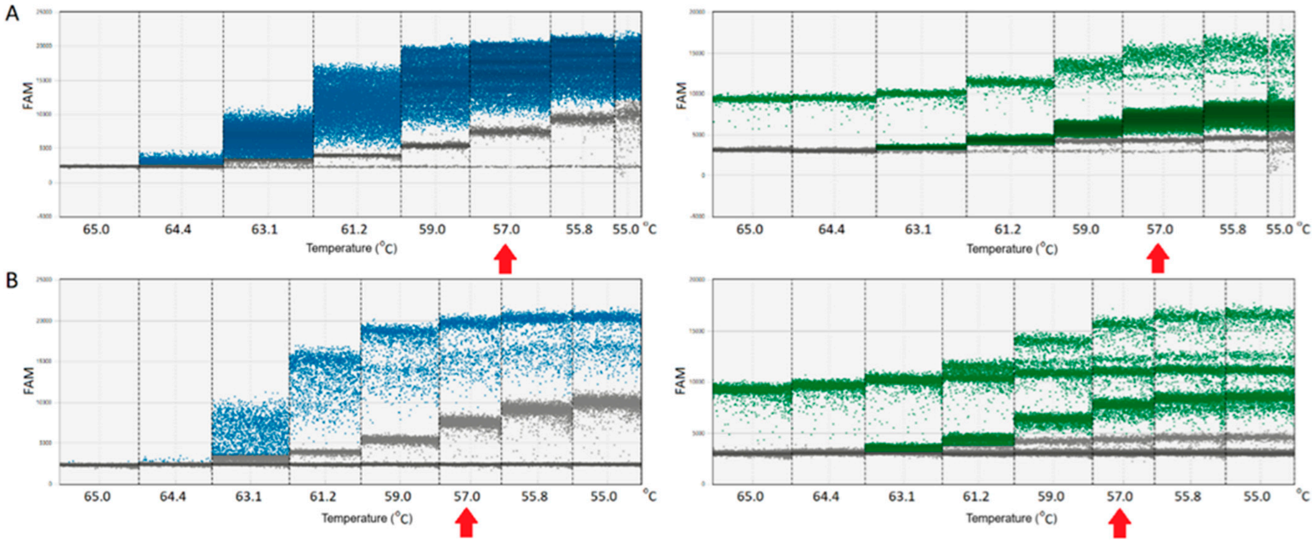

Figure S1. *Optimal annealing temperature selection.* One dimensional plots of ddPCR reactions showing an annealing temperature gradient ranging from 65°C to 55°C using cDNA derived from a HMC-1.2 10^{-3} dilution (A) and a patient with systemic mastocytosis (B). Results from *KIT* D816V mutant (blue FAM channels), *KIT* wild-type (green HEX-LOW channels) and *ABL1* (green HEX-HIGH channels) are shown. The results with the selected annealing temperature of 57°C are indicated with a red arrow.

2. KIT D816V droplet digital PCR protocol

The KIT D816V ddPCR protocol was applied for the analytical validation experiments, including the assessment of analytical sensitivity and specificity, and for testing SM patient samples. All primers and probes were ordered as HPLC purified and lyophilized. Before first use, primers and probes were dissolved in nuclease free water in a concentration of 100 µM, aliquoted and stored at -20°C. For reaction setup, all reagents except FastDigest HindIII (Thermo Fisher Scientific, Bleiswijk, the Netherlands, catalogue number FD0505, lot number 2919157, which was added directly from the -20°C freezer) were thawed, mixed and brought to room temperature. For the cDNA assay, 20 µL master mix, including the ddPCR Multiplex Supermix (Bio-Rad, Hercules, CA, USA, catalogue number 12005910), *KIT* forward and reverse primers, the *KIT* D816V and wild-type probes, the *ABL1* assay mix, and DEPCH_2O was pipetted per well in a 96 well PCR plate (Table S2). The 15.5 µL gDNA ddPCR reagent master Supermix contained all reagents, including the ddPCR Supermix (Bio-Rad, Hercules, CA, USA, catalogue number 1863023),

HindIII (gDNA assay), all primers and probes and DEPCH_2O, except the sample gDNA template. Subsequently, 5 µL cDNA or 9.5 µL gDNA was added to the wells, after which the plate was sealed using aluminum foil, vortexed and centrifuged shortly to collect the contents to the bottom of the wells. Twenty µL of PCR mix and 70 µL droplet generating oil were added to the corresponding wells of a ddPCR droplet generation cartridge and 0,795 nL sized droplets were generated with a QX200 droplet generator (Bio-Rad, Hercules, CA, USA), yielding a mean number of 18712 accepted droplets (SD: 1546) per well. Next, 40 µL of droplets were transferred to ddPCR plates, sealed with ddPCR plate seal using an automated pate sealer (PX1 PCR sealer, Bio-Rad, Hercules, CA, USA) and plates were transferred to a deep well C1000 Touch thermal cycler (Bio-Rad, Hercules, CA, USA). Cycling was performed using the following program: 1 cycle at 95 °C for 10 minutes (initial denaturation), 40 cycles at 94°C for 30 seconds (denaturation), 57°C for 1 minute (annealing and elongation) and finally followed by one cycle at 98 °C for 10 minutes (final denaturation). The temperature ramping rate was set at 2°C/second. Following PCR, the plates were analyzed within 24 hours with a QX200 Droplet Reader (Bio-Rad Laboratories, Hercules, CA, USA).

Results were analyzed using QX manager or Quanta Soft Analysis Pro software using the amplitude multiplex option (Bio-Rad Laboratories, Hercules, CA). The *KIT* D816V mutation signal was measured in the FAM-HIGH channel, the *KIT* wild-type signal in the HEX-LOW channel and the *ABL1* reference gene signal in the HEX-HIGH channel. Thresholds were manually defined by visual inspection of the 2D plots (showing clear separation of the populations). The results were expressed as a fraction (%) normalized to the *ABL1* reference gene expression or as a fraction (%) normalized to the total *KIT* expression when cDNA was used and as variant allele frequency (VAF%; percentage mutated *KIT* alleles/total *KIT* alleles) when genomic DNA was used.

To guarantee a sufficiently high assay input, the ddPCR measurements were performed in duplicate wells and results were merged and acceptable when at least 32,000 cDNA copies of the *ABL1* reference gene or 30,000 gDNA copies of *KIT* wild-type reference gene were present. When less copies were found, the analysis was repeated with more cDNA input and/or using four instead of duplicate wells. Furthermore, to avoid Poisson law estimation errors caused by too little negative droplets, samples were only accepted when at least 100 negative droplets

were present. When oversaturation with less than 100 empty droplets was found, the analysis was repeated with reduced cDNA input. Positive HMC-1.2 1:100 dilution control samples and negative No Template Control (NTC) samples, containing no cDNA or gDNA, as well as negative NBM) No Amplification Control (NAC) samples were included in all ddPCR experiments. Experiments were approved if the HMC-1,2 control was strong positive, the NTC control negative and the NAC values were below the LoB.

Table S2: DdPCR reagent master mixes for cDNA and gDNA samples.

**Multiplex cDNA assay**		
**4x ddPCR Multiplex Supermix**	6.25 µL	(Bio-Rad)
**KIT forward primer**	1.20 µL	(25 pmol/µL, Eurofins Genomics)
**KIT reverse primer**	1.20 µL	(25 pmol/µL, Eurofins Genomics)
**KIT D816V FAM probe**	0.06 µL	(100 pmol/µL, Eurofins Genomics)
**KIT wild type HEX-low probe**	0.06 µL	(100 pmol/µL, Eurofins Genomics)
**ABL1 HEX-high assay mix (stock)**	2.00 µL	(Bio-Rad)
**DEPCH2O**	9.25 µL	
**cDNA**	5.00 µL	(25 ng/ µL)
**Total**	25.00 µL	
**gDNA assay**		
**2x ddPCR Supermix**	12.50 µL	(Bio-Rad)
**KIT primers/probes mix**	1.25 µL	(Bio-Rad)
**HINDIII**	1.25 µL	(Thermo Fisher)
**DEPCH2O**	0.50 µL	
**gDNA**	9.50 µL	(50 ng/ µL)
**Total**	25.00 µL	

3. Sample collection, RNA and gDNA isolation and cDNA synthesis

For analytical validation experiments, RNA and gDNA from the human mast cell line 1.2 (HMC-1.2), harboring a heterozygous *KIT* D816V mutation, and normal bone marrow (NBM) samples from healthy BM transplantation donors were used. The HMC-1.2 cell line was obtained from Sigma-Aldrich, St. Louis, MO (reference: SCC062). For testing diagnostic relevance, samples from 79 patients with a systemic mastocytosis (SM), 73 with an indolent SM (ISM) and 6 with an advanced mastocytosis (AdvSM, Table S3) and from 15 patients with a high suspicion for SM, but negative gDNA *KIT* D816V ddPCR results were selected based on availability of

flowcytometric results and split RNA and gDNA samples. All patients were included after informed consent.

Table S3. Clinical and laboratory characteristics of patients with ISM and AdvSM.

	ISM	AdvSM	P-value
**Number of patients, n**	73	6	-
**Age in years, median (range)**	49 (15–71)	64 (62–72)	>0.05
**female, n (%)**	41 (56)	3 (50)	>0.05
**Diagnosis:**			
**ASM, n (%)**	-	1 (17)	-
**MCL, n (%)**	-	1 (17)	-
**SM-AHN, n (%)**	-	4 (67)	-

ISM: indolent systemic mastocytosis; AdvSM: advanced systemic mastocytosis, ASM: aggressive systemic mastocytosis; MCL: mast cell leukemia; SM-AHN: systemic mastocytosis with associated hematological neoplasm.

PCR amplification efficiency also depends on the presence or absence of inhibitory substances, like the clinically relevant inhibitor heparin. Therefore, we only used EDTA as anticoagulant, which seems to give no adverse effect on PCR amplification efficiency, as shown previously [1]. Bone marrow aspirate (BM) samples were collected in EDTA tubes (Greiner Bio-One GmbH, Kremsmünster, Austria) and stored at 4°C for a maximum of 48 hours before white blood cell isolation by ammonium chloride erythrocyte lysis from where the samples were split into aliquots. RNA was isolated using TriZol (Thermo Fisher Scientific, Bleiswijk, the Netherlands) according to the protocol provided by the manufacturer (method based upon a phenol/chloroform extraction) and dissolved in 20 µL nuclease free water. Genomic DNA was isolated with the QIAamp DNA Blood Mini QIAcube Kit using a QiaCube system (Qiagen, Hilden, Germany) and eluted in 100 µL $T_{10}E_1$ buffer (pH 8.0) according to the instructions of the manufacturer. Extraction blanks for RNA and gDNA were included in each run. RNA and gDNA were quantified using a NanoDrop 2000 Spectrophotometer (Thermo Fisher Scientific Inc, The Netherlands). RNA quality was approved with spectrophotometry if the OD260 nm/OD280 nm ratio was

between 1.6 and 2.0 and clearly visible 28S and 18s bands were present using RNA screentape on an Agilent 4150 Tapestation gel electrophoresis system (Agilent Technologies, Middelburg, the Netherlands). DNA quality was approved if the OD260 nm/OD280 nm ratio was between 1.8 and 2.0. Extracted gDNA was diluted to 50 ng/μL, using $T_{10}E_1$ buffer (pH 8.0) and stored at -20 °C. RNA was diluted to 170 ng/μL with nuclease free water and stored at -80°C. cDNA synthesis was performed on 1 μg RNA in a total volume of 40 μL with 1 μL SuperScript™II RNAse H^- Reverse Transcriptase, Invitrogen, Waltham, Massachusetts, USA) using random hexamer priming according to the manufacturer's instructions (Table S4). Furthermore, because we noted before that remaining traces of RNA in cDNA samples caused false positive signals [1], we included an extra final incubation step of 5 minutes at 95 °C to hydrolyze the remaining interfering RNA molecules. RNA samples were found completely negative for traces of genomic DNA. Real-Time qPCR measurements of house-keeping *Porphobilinogen deaminase* (*PBGD*) gene controls and negative $DEPCH_2O$ controls were included in all cDNA synthesis procedures to exclude inhibitory substances and contaminations, respectively. Experiments were approved if there were no positive $DEPCH_2O$ signals and *PBGD* results had Ct values below 29 and differences between duplicate measurements smaller than 2 Cq. The cDNA samples were stored at -20 °C before use.

**Table S4. cDNA reagent master mix for cDNA synthesis.**		
**cDNA reaction per sample:**		**Manufacturer, catalogue number**
5 x First Strand Buffer	8.0 μl	Invitrogen, Y02321
DTT	0.4 μl (0.1 M)	Invitrogen, Y00147
dNTPs ATP, CTP, GTP and UTP	1.6 μl (25 mM)	Fisher Scientific BV, R0481
Random hexamer	0.8 μl (5 g/L)	Roche, 11034731001
Superscript II	1.0 μl (200 U/μl)	Invitrogen, 100004925
RNAsin	1.0 μl (40 U/μl)	Promega, N2511
$DEPCH_2O$	7.2 μl	
RNA (denatured)	20 μl (50 ng/μl)	
Total	40 μl	

4. Method comparison between ddPCR and qPCR

We compared the performances of the ddPCR and a qPCR previously used for routine clinical diagnostics [2,3]. Tenfold cDNA dilution series of the HMC-1.2 cell line (carrying a heterozygous KIT D816V mutation), diluted in normal BM (NBM) samples cDNA spanning a 10^{-2} to 10^{-7} dilution range were measured six times in duplicate across six days with both assays. Figure S2 shows the log values of the ddPCR ABL1-normalized KIT D816V expression levels and the qPCR KIT D816V expression levels. While the ddPCR results showed the expected values up to a 10^{-6} dilution with a slope of 0.999 and optimal R^2 of 0.99, the qPCR analysis exhibited positive values up to a 10^{-5} dilution with a slope of 3.130, R^2 of 0.96 and mean inter-assay variation of 0.98 Ct (range: 0.73-2.09 Ct). Overall, a good concordance between the results of the ddPCR and previously used qPCR was observed, although the qPCR demonstrated one-log lower dynamic range, lower linearity and higher inter-assay variation.

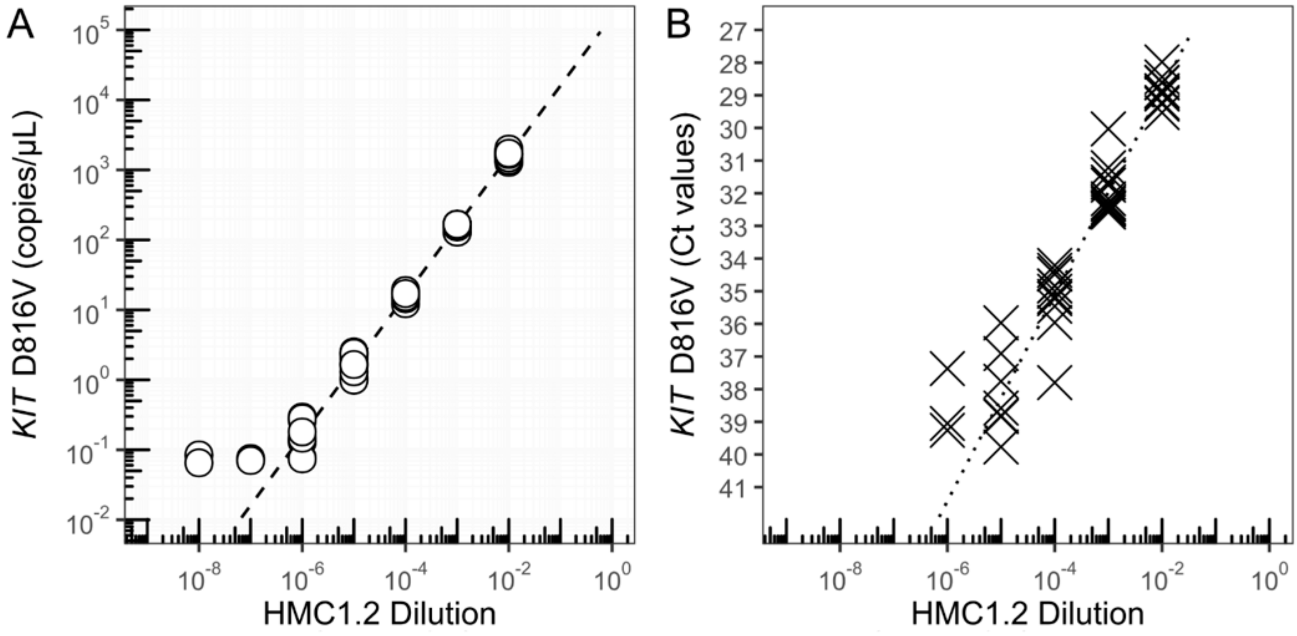

Figure S2. *Method comparison between ddPCR and qPCR*. DdPCR-derived *ABL1*-normalized *KIT* D816V expression levels (A) were compared with qPCR-derived *KIT* D816V expression values (B) using serial ten-fold HMC-1.2 cell line cDNA dilutions in NBM cDNA. The log values of the ddPCR results (copies/µL, A) and the Ct (cycle threshold) values of the qPCR (B) are

plotted against the log values of the HMC-1.2 serial cDNA dilutions. The dashed (A) and dotted (B) line represent trendlines.

References

1. Wierenga, A.T.J.; Hesp, L.B.; Simpelaar, A.; Morsink, L.M.; Woolthuis, C.M.; Schuringa, J.J; Minovic, I.; Huls, G.; Mulder, A.B. Validation of a Multiplex mRNA- and gDNA-Based Droplet Digital PCR Assay in Acute Myeloid Leukemia Patients with an NPM1 Mutation. *Clin. Chem.* **2026**, *72*, 281–290.
2. Onnes, M.C.; Alheraky, A; Nawijn, M.C.; Sluijter, T.E.; Mulder, A.B.; Arends, S.; Oude Elberink, H.N.G. Detection of clonal mast cell disease in wasp venom allergic patients with normal tryptase. *Clin. Transl. Allergy* **2022**,12, e12174.
3. Lawley, W. ; Hird, H.; Mallinder, P.; McKenna, S.; Hargadon, B.; Murray, A.; Bradding, P. Detection of an activating c-kit mutation by real-time PCR in patients with anaphylaxis. *Mutat. Res*. **2005**, *572,* 1–13.
